# The Evolving Role of FDG–PET in Behavioral Variant Frontotemporal Dementia: Current Applications and Future Opportunities

**DOI:** 10.3390/ijms262010090

**Published:** 2025-10-16

**Authors:** Serafeim Ioannidis, Natalia Konstantinidou, Alexandros Giannakis, Chrissa Sioka, Panagiotis Ioannidis

**Affiliations:** 1 School of Medicine, Aristotle University of Thessaloniki, 541 24 Thessaloniki, Greece; ioannidissera@gmail.com; 2 2nd Department of Neurology, AHEPA University Hospital, Aristotle University of Thessaloniki, 546 36 Thessaloniki, Greece; nataliak95@gmail.com (N.K.);; 3 Department of Neurology, Faculty of Medicine, School of Health Sciences, University of Ioannina, University Campus, 455 00 Ioannina, Greece; 4 Department of Nuclear Medicine, School of Health Sciences, University of Ioannina, University Campus, 455 00 Ioannina, Greece

**Keywords:** behavioral variant, frontotemporal dementia, FDG–PET, diagnostic biomarker, early diagnosis, brain hypometabolism, brain connectome, brain metabolic networks

## Abstract

The diagnosis of behavioral variant of frontotemporal dementia (bvFTD)—a common cause of early-onset dementia—remains challenging due to a lack of determined biomarkers. 18F-fluorodeoxyglucose-positron emission tomography (FDG–PET) scan detects early glucose metabolism alterations in specific brain regions. The detection of distinct hypometabolic patterns in early stages of bvFTD has established FDG–PET as an indispensable adjunctive diagnostic tool in inconclusive cases, as well as in distinguishing between different types of dementia. Moreover, its role in the differential diagnosis of the often overlapping bvFTD and primary psychiatric disorders (PPD) is being studied by exploring disease-specific hypometabolic areas. Finally, the identification of early metabolic alterations and even earlier alterations in distinct metabolic brain networks may assist the diagnosis of presymptomatic carriers of disease-related gene mutations and lead to the development of novel biomarkers. The aim of our review is to underscore the role of FDG–PET as an approved yet promising tool that may lead to a new era in the diagnosis of bvFTD by establishing novel biomarkers and integrating AI as an assistant modality to inform diagnosis and decision-making.

## 1. Introduction

Behavioral variant of frontotemporal dementia (bvFTD) is a neurological disorder characterized by progressive decline primarily in the patient’s socioemotional and executive functions [1]. The above syndrome accounts for approximately half of the cases of a larger group of neurodegenerative disorders called frontotemporal dementia (FTD), which share common frontotemporal lobar degeneration (FTLD) pathology [2]. The clinical subtypes of this entity include the following: (1) the bvFTD, (2) the semantic variant of primary progressive aphasia (svPPA), and (3) the non-fluent variant of PPA (nfvPPA) [3].

BvFTD is the most common FTD syndrome, predominantly affecting middle-aged individuals, with peak prevalence in the early sixties [4]. The majority of cases are sporadic, but a causative autosomal-dominant genetic mutation may be found in almost 20% of the cases [5,6]. Its diagnosis constitutes a real challenge for clinicians, since, in early stages, short-term memory and visuospatial functions are usually spared, and the initial manifestations concern mostly behavioral changes that may easily be overlooked or mistaken for a psychiatric disorder [7]. The core clinical features that guide the diagnosis are as follows: (1) apathy and loss of motivation, (2) behavioral disinhibition, (3) loss of sympathy or empathy, (4) ritualistic or stereotyped behaviors, (5) hyperphagia and dietary changes, and (6) executive deficits. Even though minor or major issues may arise in their personal, social, or professional life, patients themselves rarely complain about any symptoms [8]. Thus, caregivers have the leading role in obtaining a thorough and reliable clinical history, which is the basis for reaching a correct diagnosis.

Apart from clinical examination, history, and neuropsychological studies, brain imaging is crucial to establish a probable diagnosis of the syndrome and exclude other causes of dementia (either neurodegenerative or not) [8]. Structural magnetic resonance imaging (MRI) is the first-line imaging modality in order to assess the atrophy pattern and rule out other possible diagnoses [9]. However, functional imaging may be useful, especially in inconclusive cases, by revealing brain abnormalities—such as hypometabolism or hypoperfusion—long before structural atrophy is evident [10]. The gold standard of functional brain imaging in the diagnosis of bvFTD is an 18F-fluorodeoxyglucose-positron emission tomography (18F FDG–PET) scan, due to its high diagnostic accuracy, when combined with MRI studies [11].

An 18 FDG–PET scan uses fludeoxyglucose as a radiotracer in order to identify tissues with altered glucose metabolism. Since the brain’s main source of energy is glucose, FDG–PET can detect patterns of glucose hypometabolism that are present in different neurodegenerative disorders, even prior to structural changes [12]. Therefore, an 18F FDG–PET scan constitutes a valuable tool in timely diagnosis, as well as in differential diagnosis of bvFTD [13]. In addition, Rascovsky’s diagnostic criteria for bvFTD support the use of the aforementioned functional neuroimaging technique in order to upgrade the diagnosis from possible to probable [8].

In this review article, we aim to determine the role and highlight the importance of FDG–PET in the diagnosis of bvFTD, while investigating new aspects of its use that may challenge the status quo and pave the way for a new era in the approach to this clinical entity.

## 2. Methods

A search of the MEDLINE and Scopus databases was conducted using the keywords “18F-FDG-PET,” “glucose metabolism,” “frontotemporal dementia,” “bvFTD,” and “FTLD.” Additional relevant studies were retrieved from the already identified articles. Only full-text articles published in English were included. The search initially yielded 831 records. After removing duplicates and excluding irrelevant studies, 56 articles were included in the final analysis.

## 3. Results

### 3.1. The Established Role of 18F FDG–PET SCAN as a Diagnostic Tool for bvFTD

Clinical history, physical and neurological examination, neuropsychological assessment, and specific clinical scales and cognitive tests form the initial approach to a patient with suspected bvFTD, since they help identify the clinical syndrome [1]. However, according to Rascovsky’s diagnostic criteria for bvFTD, apart from functional decline, neuroimaging studies are indispensable in order to reach a probable bvFTD diagnosis [8]. Structural imaging through MRI studies is the first step to confirm the diagnosis by excluding other etiologies and detecting patterns of brain atrophy typical of bvFTD (medial frontal, orbital–insular and anterior temporal cortical regions) [14]. Brain atrophy, though, may sometimes not be apparent in the early stages of the disease [15]. In these cases, functional neuroimaging is recommended as a useful adjunctive diagnostic tool. Therefore, an 18F FDG–PET scan, with its proven advantage over single photon emission tomography (SPECT), is the next step in cases of diagnostic uncertainty [11].

Similarly to typical brain atrophy patterns in structural MRI, patterns of glucose hypometabolism specific for bvFTD can be identified in FDG–PET scan. The regions predominantly affected are the medial frontal, dorsolateral, and anterior temporal cortices [16,17,18]. However, other brain areas may also participate in the hypometabolic profile of certain bvFTD patients, especially in later stages of the disease or genetic forms of bvFTD. Nevertheless, the involvement of anterior cingulate and anterior temporal regions raises the likelihood of a bvFTD diagnosis [19]. Atypical patterns of hypometabolism that have been reported concern mostly bvFTD related to specific gene mutations or bvFTD/motor neuron disease (MND), and involve mainly frontal, pure temporal, parietal areas or even a normal 18F FDG–PET scan. These cases may also have atypical initial manifestations with psychosis and benign, slow clinical deterioration, together with a normal scan [10,20,21,22,23,24].

European Association of Nuclear Medicine and European Academy of Neurology released in 2018 specific recommendations for the use of 18F FDG–PET scan in neurodegenerative cognitive disorders, including FTLD [25]. Firstly, experts suggest its use on the basis of detecting brain hypometabolism in the initial stages of bvFTD.

Additionally, 18F FDG–PET is a necessary adjunctive tool to the standard approach (clinical, neuropsychological, and MRI assessment) for differentiating bvFTD from other neurodegenerative causes of dementia [26]. More evidence exists to support the use of FDG–PET in the differential diagnosis of bvFTD and Alzheimer’s disease (AD), where sensitivity ranges from 80 to 99%, specificity from 63 to 98% and accuracy from 87 to 89.2% [11,22,27,28]. In AD, the affected areas concern mostly the temporoparietal regions, especially the precuneus and posterior cingulate cortex. Although in the majority of cases the patterns of hypometabolism in bvFTD and AD are clearly distinct, sometimes frontoparietal regions may be affected in both clinical entities. In such cases, cerebrospinal fluid biomarkers and amyloid PET can be proven valuable in distinguishing bvFTD from AD [14].

Moreover, clinical overlap may sometimes be present between dementia with Lewy bodies (DLB) and bvFTD, especially in the presence of Parkinsonism [29,30]. Regarding the patterns of hypometabolism revealed by the 18F FDG–PET scan, DLB involves primarily the occipital and temporoparietal areas, while the posterior cingulate cortex is spared (cingulate island sign). On the other hand, as mentioned above, in bvFTD, frontal and anterior temporal areas are hypometabolic. Hence, Nobili et al. in 2018 [25] recommended the use of 18F FDG–PET in the differential diagnosis of these two entities, based on the different patterns of hypometabolism and the inclusion of the modality in the diagnostic criteria of both bvFTD and DLB.

### 3.2. Novel Applications of 18F FDG–PET Scan in bvFTD

#### 3.2.1. Early Detection of Preclinical bvFTD in Asymptomatic Mutation Carriers

Early metabolic changes, as measured by 18F FDG–PET, represent a promising biomarker for the timely diagnosis and monitoring of bvFTD, particularly in asymptomatic carriers of relevant mutations. A growing body of evidence indicates that functional alterations can be detected years, and in some cases more than a decade, before the onset of overt clinical symptoms [31,32,33,34,35,36,37,38,39]. This preclinical window offers a critical opportunity for early diagnosis and future therapeutic intervention. The three most common mutations associated with familial bvFTD are *MAPT*, *GRN*, and *C9orf72* [40].

In *MAPT* mutation carriers, FDG–PET studies have provided important insight into early regional vulnerability. In carriers of the intron 10 + 3 mutation, comparisons of symptomatic and asymptomatic individuals revealed that while MRI showed significantly greater atrophy in symptomatic carriers compared to asymptomatic ones, FDG–PET did not show statistically significant differences in hypometabolism between the two groups, suggesting that functional impairment emerges before extensive structural degeneration. The most prominent changes were observed in the medial temporal lobes and limbic system [31]. Another study of carriers of the P301L mutation, imaged on average 12.5 years before expected onset, identified reduced metabolism restricted to the anterior cingulate cortex. Importantly, this finding aligned with structural MRI changes, highlighting the anterior cingulate as a possible site of early neurodegeneration in this mutation. Whether hypometabolism or atrophy is the first measurable abnormality in this region remains unclear, but both converge on its pivotal role in disease initiation [32].

Further work has highlighted that in *MAPT* carriers, early functional changes may not always manifest as widespread hypometabolism, but instead as altered network organization. For example, one study reported hypometabolism in the triangular part of the inferior frontal gyrus alongside hypermetabolism in the orbital part of the same region, without significant metabolic differences in the anterior cingulate cortex. Nevertheless, network analyses revealed that the anterior cingulate acted as a “lost hub,” reflecting a disrupted functional circuit. Additional hypofunctioning regions included the ventromedial prefrontal cortex and orbitofrontal cortex, both located within the medial prefrontal lobe. In contrast, the default mode network (DMN) and salience network (SN) were paradoxically overactivated. This pattern may represent compensatory upregulation, as the brain attempts to preserve global connectivity despite ongoing focal dysfunction. Importantly, such compensatory mechanisms appear to collapse once clinical symptoms emerge, at which point reduced connectivity within the DMN and SN becomes apparent [33].

A complementary study focusing on the insula in the same population subdivided this structure into functional regions of interest. Although no significant structural or metabolic changes were observed relative to controls, connectivity alterations were clearly present in asymptomatic carriers. Specifically, connectivity between the anterior insula and the orbitofrontal cortex and dorsolateral superior frontal gyrus was increased, whereas connections with the middle temporal gyrus and temporal poles were reduced. In patients with manifest bvFTD, the anterior insula exhibited both gray matter atrophy and hypometabolism, demonstrating that early network-level reorganization precedes measurable regional degeneration. Together, these findings suggest that altered connectivity may serve as a more sensitive early biomarker than regional metabolism alone, and that the breakdown of brain network equilibrium plays a central role in the pathogenesis of bvFTD in *MAPT* mutation carriers [34].

Similar insights have been gained from the study of *GRN* mutation carriers. 18F FDG–PET reveals early and asymmetric functional alterations, often well in advance of structural changes. In both asymptomatic individuals and those classified as cognitively symptomatic but not demented (CSND), hypometabolism was consistently observed in the frontal and anterior temporal lobes, with a predominance in the right hemisphere [35]. Moreover, in another study that excluded CSND patients, the left middle temporal gyrus proved to be a particularly important region, showing significant hypometabolism in asymptomatic carriers despite the absence of detectable atrophy on MRI. Longitudinal follow-up MRI scans, conducted 20 months apart, confirmed this early vulnerability, demonstrating maximal atrophy in the same left temporal region that had previously shown metabolic decline. This sequence suggests that 18F FDG–PET can capture functional changes preceding, and likely driving, subsequent structural degeneration [36].

The role of the middle and superior temporal gyri in *GRN*-related disease is further underscored by a larger longitudinal study. In a five-year follow-up of 27 mutation carriers, hypometabolism was consistently identified in the left temporal lobe, particularly the middle and superior temporal gyri, even in presymptomatic individuals. Notably, the greatest rate of metabolic decline over time shifted contralaterally to the right temporal lobe, illustrating the asymmetric progression characteristic of *GRN*-associated disease. According to the study’s results, these early metabolic alterations were detectable more than 15 years before the estimated age of symptom onset, while MRI revealed no significant atrophy even at the final follow-up. Thus, 18F FDG–PET provides a uniquely sensitive window into the earliest phases of *GRN*-related bvFTD [37].

In *C9orf72* mutation carriers, early metabolic abnormalities appear more diffuse and widespread. 18F FDG–PET studies involving asymptomatic carriers consistently show hypometabolism across multiple frontotemporal regions, along with bilateral thalamic involvement. The consistent presence of thalamic hypometabolism suggests that subcortical structures may play a key role in the earliest pathophysiological processes associated with this mutation. Additional hypometabolic changes have been described in the inferior parietal lobes, while regions of hypermetabolism have also been reported, including the precentral gyrus, superior frontal gyrus, and precuneus. As in *MAPT* carriers, such hypermetabolism may represent compensatory mechanisms aimed at maintaining functional connectivity during the presymptomatic stage [38,39].

#### 3.2.2. Metabolic Brain Networks as a Novel Biomarker

Over the past decade, 18F FDG–PET has been increasingly employed to investigate the integrity of large-scale neural networks in patients with FTD. Network alterations generally parallel regional hypometabolism, with predominant involvement of the frontal and temporal lobes, whereas the parietal lobes are typically spared [41].

In the bvFTD, two networks have been identified as central to disease pathology: the default mode network (DMN) and the salience network [42]. The DMN, normally engaged during resting states, subserves functions that are characteristically impaired in bvFTD, such as theory of mind and social cognition. In contrast, the salience network is activated by external stimuli, suppresses DMN activity, and redirects attentional resources toward goal-directed behavior [43]. Both networks show marked disruption in bvFTD. Importantly, the anterior DMN is preferentially impaired in bvFTD, whereas the posterior DMN is predominantly affected in AD, providing a potential neuroimaging marker to aid differential diagnosis [44].

Recent 18F FDG–PET studies have further delineated bvFTD subtypes based on distinct patterns of network dysfunction. The frontal variant, characterized by predominant prefrontal hypometabolism, demonstrates abnormally increased anterior DMN activity accompanied by mild salience network involvement. By contrast, the temporo-limbic variant, associated with predominant temporal lobe hypometabolism and relative preservation of the prefrontal cortex, exhibits reduced anterior DMN connectivity alongside pronounced salience network dysfunction [45].

The investigation of brain metabolic networks derived from PET imaging has enabled the identification of a quantitative biomarker, the bvFTD-specific multivariate metabolic brain pattern (bFDRP), which is consistently elevated in patients with bvFTD. This pattern distinguishes bvFTD from healthy controls and other dementia types with high accuracy, and its reproducibility has been confirmed across multiple cohorts. Although disruptions in the default mode network (DMN) are observed in bvFTD, they are not disease-specific, as partial hypometabolism can occur in other conditions. In contrast, the bFDRP encompasses bvFTD-specific hypometabolic regions beyond the DMN and exhibits pronounced network disconnection, rendering it a more reliable and specific biomarker for bvFTD, even when posterior DMN connectivity remains relatively preserved [46,47]. Nevertheless, the implementation of the bFDRP in clinical practice remains to be established, as its use to date has been confined to research settings.

Recent research has increasingly focused on leveraging brain network analysis to develop novel biomarkers for neurodegenerative diseases. A unique, recently published study applied a multiplex connectomics approach, integrating both cortical thickness (CTH) and 18F FDG–PET within a dual-layer model. This framework allowed direct comparison of the two modalities and enabled calculation of the multiplex participation coefficient (MPC), a measure of how similarly regions are represented across structural (CTH) and metabolic FDG–PET networks. Higher MPC values indicate greater concordance between the two modalities.

The study demonstrated that this multiplex method identified network disruptions in regions consistent with findings from traditional single-modality (“unilayer”) approaches, supporting its reliability. By combining structural and metabolic information, multiplex connectomics provides a more comprehensive picture of network alterations and represents a promising avenue for the development of future diagnostic and prognostic biomarkers [48].

### 3.3. Differentiating bvFTD from Primary Psychiatric Disorders

Perhaps the greater challenge in the differential diagnosis is distinguishing between bvFTD and late-onset primary psychiatric disorders (PPD), since a number of PPD may present with behavioral changes mimicking bvFTD, such as apathy, compulsiveness, and social dysfunction [49]. Late-onset major depressive disorder, bipolar disorder, schizophrenia, obsessive–compulsive disorder, autism spectrum disorders, and personality disorders may sometimes be incorrectly diagnosed as bvFTD [50]. This clinical overlap is actually translated into numbers, since 50–71% of bvFTD cases are initially diagnosed as a psychiatric disorder, resulting in almost a 6-year delay of the correct diagnosis of bvFTD [7,51,52,53]. As a consequence, patients do not receive timely and appropriate management and treatment nor the right information regarding the course and prognosis of their condition. De Boer et al. [54] reported diagnostic instability in a 2-year follow-up period, with more than 20% of the late-onset frontal lobe (LOF) study population switching diagnosis. After that period of time, a small number of study subjects had an alternative final diagnosis, thus establishing 2 years as a safe period of time to determine a bvFTD diagnosis.

In 2020, the Neuropsychiatric International Consortium for Frontotemporal Dementia composed clear recommendations regarding the clinical approach of differential diagnosis between bvFTD and PPD [55]. Clinical evolution and functional decline over 1 year, familial history of neuropsychiatric disorders, and motor neuron or extrapyramidal signs point towards a bvFTD rather than a PPD diagnosis. According to Ducharme et al., social cognition assessment should be integrated into the standard neuropsychological battery for bvFTD. A recent study by van Engenel et al. [56] reveals a higher score on facial emotion recognition in phenocopy syndrome of bvFTD (phFTD) patients—a clinically bvFTD-like syndrome lacking neuroimaging abnormalities and clinical progression [57]—compared to bvFTD patients. Moreover, the Consortium supported the use of CSF or serum NfL as a biomarker of neurodegeneration [58,59,60], along with the screening for the C9orf72 mutation in all possible/probable bvFTD cases, as well as dubious cases with a strong psychiatric component that do not fulfill the bvFTD diagnostic criteria [61].

It is common knowledge that slight structural or metabolic abnormalities in shared frontotemporal regions may be found on MRI or FDG–PET scan in certain PPD and bvFTD [53]. This fact, along with the aforementioned clinical overlap between bvFTD and PPD, may negatively affect the diagnostic accuracy of neuroimaging in bvFTD. Vijverberg et al. [62], in 2016, studied and determined the diagnostic accuracy of MRI, adjunctive 18F FDG–PET, and their combination in a heterogeneous population with late-onset behavioral changes. The study concluded in moderate sensitivity and high specificity of MRI studies for bvFTD. On the other hand, an additional 18F FDG–PET scan showed high sensitivity but relatively low specificity due to false-positive cases, as PPD may also be characterized by synaptic dysfunction in some brain regions. The combination of the two modalities presented high sensitivity and suboptimal specificity. In 2020, the Consortium proposed incorporating a standardized review protocol with validated visual atrophy rating scales, as well as volumetric analyses, into high-resolution 3D-T1 brain MRI sequences. As far as the use of FDG–PET is concerned, a normal scan can rule out bvFTD, while an atypical hypometabolic pattern should not be over-interpreted, when a psychiatric alternative diagnosis is possible [55].

The value of 18F FDG–PET in excluding a neurodegenerative cause is also confirmed by the recent study of Hellwig et al. among patients with depression and cognitive decline [63]. FDG–PET increased the accuracy of diagnosis in the study population by 20% in differentiating between neurodegenerative and non-neurodegenerative etiologies, as well as in distinguishing between different neurodegenerative causes of dementia. Former studies support as well the utility of 18F FDG–PET in discriminating between bvFTD and PPD or phFTD [10,64].

However, Vijverber et al. reported a large percentage of false-positive PPD cases, in which a bilateral frontotemporal or—in fewer cases—parietal hypometabolism was found [62]. Moreover, in Cagni et al.’s PET/MRI study [24], hypometabolism in the dorsolateral and orbitofrontal regions and caudate detected in bvFTD seems to raise diagnostic accuracy. It is also reported that slight hypometabolism in the anterior cingulate, superior parietal, and superior temporal areas may be present in genetic bvFTD or late-onset PPD, and thus may not aid in differential diagnosis. In another PET/MRI study, hypometabolism is described in the left orbitofrontal cortex, superior temporal gyrus, anterior cingulate cortex, and caudate in bvFTD compared to bipolar disorder [65].

Van Engelen et al., in their recent study in 2023 [56], suggest the use of voxel-wise comparisons of 18F FDG–PET images in order to recognize different hypometabolic profiles between bvFTD and PPD. They indicate different patterns of hypometabolism comparing the two clinical entities. In bvFTD patients, reduced metabolism was observed in the dorsal anterior cingulate cortex (dACC), insula, Broca’s area, caudate, thalamus, orbitofrontal cortex, and temporal cortex, whereas PPD patients showed motor cortex hypometabolism. Symptom severity, such as social and executive dysfunction and compulsiveness, was associated with the degree of hypometabolism in dACC and frontotemporal regions. Finally, dACC was proposed as a key hypometabolic area for discriminating between bvFTD and PPD.

## 4. Discussion

**Figure 1:**
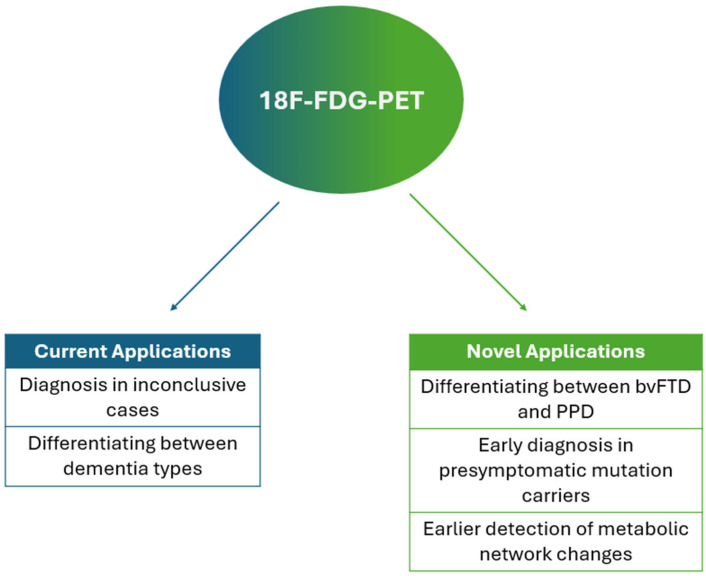
Current (blue) and future (green) applications of FDG–PET in the diagnosis of bvFTD.

This review summarizes the current clinical application of 18F FDG–PET in daily practice and, more importantly, highlights its potential future applications based on the latest research. We present the existing evidence with a particular focus on three promising areas of FDG PET in the context of bvFTD: (1) preclinical disease detection and the possibility of initiating therapy at an earlier stage in individuals carrying bvFTD-related mutations, (2) differential diagnosis between primary psychiatric disorders (PPD) and bvFTD, and (3) mapping of the brain’s connectome to enable even earlier detection of disease-related changes. Our goal is to emphasize the yet unrealized potential of FDG PET as an even more powerful diagnostic tool in bvFTD (Figure 1).

Since the first systematic investigation of FDG–PET patterns in FTD patients in 1998, numerous studies have demonstrated its utility in the differential diagnosis of various dementia types, establishing it as a first-line tool for clarifying inconclusive cases [66]. Hypometabolism is typically observed in the medial frontal, dorsolateral frontal, and anterior temporal cortices, and often precedes detectable structural atrophy on MRI [16,17,18]. Indeed, metabolic alterations can appear so early that patients may remain asymptomatic. However, the high cost of PET and its limited availability, particularly in certain healthcare systems, preclude its use as a screening tool for the general population. Nevertheless, FDG–PET may be particularly valuable in high-risk individuals, such as those with a family history of bvFTD who carry a known bvFTD-associated mutation [10].

As already analyzed above, 18F FDG–PET provides a powerful biomarker for the detection of preclinical bvFTD. It consistently detects early, region-specific changes in glucose metabolism in presymptomatic carriers of all three major bvFTD-associated mutations. These alterations often precede detectable atrophy on MRI, underscoring the functional sensitivity of metabolic imaging [32]. Even in the absence of overt hypometabolism, network-level analyses of FDG–PET data may still reveal subtle alterations. The regions most vulnerable to early hypometabolism vary by genetic subtype: the medial temporal lobe, the inferior frontal gyrus and anterior cingulate cortex in *MAPT*, the middle and superior temporal gyri in *GRN*, and widespread frontotemporal and thalamic regions in *C9orf72*. Finally, the presence of hypermetabolism in select cortical regions across mutations suggests that compensatory mechanisms are initially engaged but ultimately fail as clinical disease manifests.

On the contrary, foreseeing the disease in mutation carriers with no evident symptoms raises significant ethical concerns, as such knowledge can profoundly alter an individual’s life by affecting relationships, future planning, and psychological well-being. The findings from these studies will gain their greatest practical value once effective therapies become available. Nevertheless, they already provide valuable insights into disease pathophysiology and the progression of neurodegeneration. A good example is the following: as mentioned above, the default mode and salience networks are heavily affected in bvFTD patients, as demonstrated by brain metabolism studies [43,44,45]. Interestingly, studies in presymptomatic MAPT mutation carriers revealed that these networks are overactivated very early in the disease course, potentially losing this compensatory balance later on [33].

More broadly, studying the brain’s connectome with 18F FDG–PET represents a recent advancement that may serve as an even earlier biomarker than regional hypometabolism, although it is not yet widely implemented. A reproducible and consistent metabolic connectivity pattern has already been proposed that could support earlier diagnosis [46]. It is essential for physicians to be aware of this development, even though further studies are required to validate the universality of this diagnostic signature. This novel use of PET is likely to play an increasingly important role in the future.

In addition to this, regarding the future, automated machine learning algorithms are expected to play a crucial role in assisting healthcare professionals in reaching accurate diagnoses by integrating results from multiple clinical and imaging examinations. Given the diagnostic value of PET in differentiating between dementia subtypes, several experimental models have been developed that utilize PET images for this purpose. Whether designed specifically to distinguish FTD from AD and healthy controls, or to differentiate among a broader range of dementia types, these models have demonstrated high accuracy—often surpassing that of healthcare professionals in direct comparisons [67,68]. Notably, most of these models rely solely on PET images, highlighting the modality’s strength as a diagnostic tool.

However, the importance of human judgment remains undeniable. For example, in a study by Perovnik et al., experienced readers achieved higher specificity—meaning fewer false-positive results—than the AI model tested, even though the latter demonstrated higher sensitivity and overall accuracy. For this reason, machine learning algorithms should be designed to support, not replace, human expertise [69].

A state-of-the-art machine learning tool called State-Viewer (An FDG-PET–Based Machine Learning Framework to Support Neurologic Decision-Making in Alzheimer Disease and Related Disorders—https://www.neurology.org/doi/10.1212/WNL.0000000000213831, accessed on 30 July 2025) has been developed for precisely this purpose: It uses only a patient’s PET image to differentiate between nine dementia subtypes with high accuracy. Notably, depending on the sensitivity or specificity thresholds set by the user, the algorithm provides up to three possible diagnoses, thereby aiding clinical decision-making. When tested, the combined decision of the user with the program achieved higher accuracy than either the user or the program alone, underscoring the value of this collaborative approach [70].

Lastly, we discussed the value of PET in differentiating bvFTD from PPD, two conditions that often present with overlapping clinical features [49]. As noted, PET plays an important role in ruling out bvFTD; however, a pathological PET finding does not necessarily exclude PPD, leading to a considerable number of false-positive diagnoses [55,62]. Current research is therefore focused on identifying disease-specific regions, such as the dorsal anterior cingulate cortex (dACC), which appears to be strongly suggestive of bvFTD and may aid in distinguishing these entities [56]. At present, however, a combined approach using both MRI and PET remains the most reliable. Further studies involving large patient cohorts directly comparing bvFTD and PPD are needed to establish robust and reproducible biomarkers.

## 5. Conclusions

FDG–PET is a vital tool in the differential diagnosis of bvFTD, not only from other dementias—its most established use so far—but also from primary psychiatric disorders. Metabolic alterations can be detected before structural changes and even before clinical symptoms emerge. Emerging evidence suggests that the investigation of brain network alterations may reveal abnormalities at an even earlier stage, preceding detectable metabolic deficits. This approach could be particularly valuable for the early identification of disease in high-risk groups, such as mutation carriers. Looking ahead, the integration of AI into clinical practice is anticipated, not as a replacement for physicians but as an adjunctive tool to support diagnosis and decision-making.

## Data Availability

Not applicable.

## References

[1] Boeve B.F. (2022). Behavioral Variant Frontotemporal Dementia. Continuum.

[2] Johnson J.K., Diehl J., Mendez M.F., Neuhaus J., Shapira J.S., Forman M., Chute D.J., Roberson E.D., Pace-Savitsky C., Neumann M. (2005). Frontotemporal lobar degeneration: Demographic characteristics of 353 patients. Arch. Neurol..

[3] Olney N.T., Spina S., Miller B.L. (2017). Frontotemporal Dementia. Neurol. Clin..

[4] Coyle-Gilchrist I.T., Dick K.M., Patterson K., Vázquez Rodríquez P., Wehmann E., Wilcox A., Lansdall C.J., Dawson K.E., Wiggins J., Mead S. (2016). Prevalence, characteristics, and survival of frontotemporal lobar degeneration syndromes. Neurology.

[5] Rohrer J.D., Guerreiro R., Vandrovcova J., Uphill J., Reiman D., Beck J., Isaacs A.M., Authier A., Ferrari R., Fox N.C. (2009). The heritability and genetics of frontotemporal lobar degeneration. Neurology.

[6] Rademakers R., Neumann M., Mackenzie I.R. (2012). Advances in understanding the molecular basis of frontotemporal dementia. Nat. Rev. Neurol..

[7] Woolley J.D., Khan B.K., Murthy N.K., Miller B.L., Rankin K.P. (2011). The diagnostic challenge of psychiatric symptoms in neurodegenerative disease: Rates of and risk factors for prior psychiatric diagnosis in patients with early neurodegenerative disease. J. Clin. Psychiatry.

[8] Rascovsky K., Hodges J.R., Knopman D., Mendez M.F., Kramer J.H., Neuhaus J., van Swieten J.C., Seelaar H., Dopper E.G.P., Onyike C.U. (2011). Sensitivity of revised diagnostic criteria for the behavioural variant of frontotemporal dementia. Brain.

[9] Soucy J.P., Bartha R., Bocti C., Borrie M., Burhan A.M., Laforce R., Rosa-Neto P. (2013). Clinical applications of neuroimaging in patients with Alzheimer’s disease: A review from the Fourth Canadian Consensus Conference on the Diagnosis and Treatment of Dementia 2012. Alzheimer’s Res. Ther..

[10] Kerklaan B.J., van Berckel B.N., Herholz K., Dols A., van der Flier W.M., Scheltens P., Pijnenburg Y.A.L. (2014). The added value of 18-fluorodeoxyglucose-positron emission tomography in the diagnosis of the behavioral variant of frontotemporal dementia. Am. J. Alzheimer’s Dis. Other Dement..

[11] Mosconi L., Tsui W.H., Herholz K., Pupi A., Drzezga A., Lucignani G., Reiman E.M., Holthoff V., Kalbe E., Sorbi S. (2008). Multicenter standardized 18F-FDG PET diagnosis of mild cognitive impairment, Alzheimer’s disease, and other dementias. J. Nucl. Med..

[12] Tai Y.F., Piccini P. (2004). Applications of positron emission tomography (PET) in neurology. J. Neurol. Neurosurg. Psychiatry.

[13] Bohnen N.I., Djang D.S., Herholz K., Anzai Y., Minoshima S. (2012). Effectiveness and safety of 18F-FDG PET in the evaluation of dementia: A review of the recent literature. J. Nucl. Med..

[14] Filippi M., Agosta F., Barkhof F., Dubois B., Fox N.C., Frisoni G.B., Jack C.R., Jahannsen P., Miller B.L., Nestor P.J. (2012). EFNS task force: The use of neuroimaging in the diagnosis of dementia. Eur. J. Neurol..

[15] Perry R.J., Miller B.L. (2001). Behavior and treatment in frontotemporal dementia. Neurology.

[16] Diehl J., Grimmer T., Drzezga A., Riemenschneider M., Förstl H., Kurz A. (2004). Cerebral metabolic patterns at early stages of frontotemporal dementia and semantic dementia. A PET study. Neurobiol. Aging.

[17] Franceschi M., Anchisi D., Pelati O., Zuffi M., Matarrese M., Moresco R.M., Fazio F., Perani D. (2005). Glucose metabolism and serotonin receptors in the frontotemporal lobe degeneration. Ann. Neurol..

[18] Jeong Y., Cho S.S., Park J.M., Kang S.J., Lee J.S., Kang E., Na D.L., Kim S.E. (2005). 18F-FDG PET findings in frontotemporal dementia: An SPM analysis of 29 patients. J. Nucl. Med..

[19] Womack K.B., Diaz-Arrastia R., Aizenstein H.J., Arnold S.E., Barbas N.R., Boeve B.F., Clark C.M., DeCarli C.S., Jagust W.J., Leverenz J.B. (2011). Temporoparietal hypometabolism in frontotemporal lobar degeneration and associated imaging diagnostic errors. Arch. Neurol..

[20] Cerami C., Dodich A., Lettieri G., Iannaccone S., Magnani G., Marcone A., Gianolli L., Cappa S.F., Perani D. (2016). Different FDG-PET metabolic patterns at single-subject level in the behavioral variant of fronto-temporal dementia. Cortex.

[21] Kipps C.M., Hodges J.R., Fryer T.D., Nestor P.J. (2009). Combined magnetic resonance imaging and positron emission tomography brain imaging in behavioural variant frontotemporal degeneration: Refining the clinical phenotype. Brain.

[22] Foster N.L., Heidebrink J.L., Clark C.M., Jagust W.J., Arnold S.E., Barbas N.R., DeCarli C.S., Turner R.S., Koeppe R.A., Higdon R. (2007). FDG-PET improves accuracy in distinguishing frontotemporal dementia and Alzheimer’s disease. Brain.

[23] Solje E., Aaltokallio H., Koivumaa-Honkanen H., Suhonen N.M., Moilanen V., Kiviharju A., Traynor B., Tienari P.J., Hartikainen P., Remes A.M. (2015). The Phenotype of the C9ORF72 Expansion Carriers According to Revised Criteria for bvFTD. PLoS ONE.

[24] Cagnin A., Pigato G., Pettenuzzo I., Zorzi G., Roiter B., Anglani M.G., Bussè C., Mozzetta S., Gabelli C., Campi C. (2024). Data-driven analysis of regional brain metabolism in behavioral frontotemporal dementia and late-onset primary psychiatric diseases with frontal lobe syndrome: A PET/MRI study. Neurobiol. Aging.

[25] Nobili F., Arbizu J., Bouwman F., Drzezga A., Agosta F., Nestor P., Walker Z., Boccardi M. (2018). European Association of Nuclear Medicine and European Academy of Neurology recommendations for the use of brain (18) F-fluorodeoxyglucose positron emission tomography in neurodegenerative cognitive impairment and dementia: Delphi consensus. Eur. J. Neurol..

[26] Nestor P.J., Altomare D., Festari C., Drzezga A., Rivolta J., Walker Z., Bouwman F., Orini S., Law I., Agosta F. (2018). Clinical utility of FDG-PET for the differential diagnosis among the main forms of dementia. Eur. J. Nucl. Med. Mol. Imaging.

[27] Poljansky S., Ibach B., Hirschberger B., Männer P., Klünemann H., Hajak G., Marienhagen J. (2011). A visual [18F]FDG-PET rating scale for the differential diagnosis of frontotemporal lobar degeneration. Eur. Arch. Psychiatry Clin. Neurosci..

[28] Rabinovici G.D., Rosen H.J., Alkalay A., Kornak J., Furst A.J., Agarwal N., Mormino E.C., O’Neil J.P., Janabi M., Karydas A. (2011). Amyloid vs. FDG-PET in the differential diagnosis of AD and FTLD. Neurology.

[29] Claassen D.O., Parisi J.E., Giannini C., Boeve B.F., Dickson D.W., Josephs K.A. (2008). Frontotemporal dementia mimicking dementia with Lewy bodies. Cogn. Behav. Neurol..

[30] Morgan S., Kemp P., Booij J., Costa D.C., Padayachee S., Lee L., Barber C., Carter J., Walker Z. (2012). Differentiation of frontotemporal dementia from dementia with Lewy bodies using FP-CIT SPECT. J. Neurol. Neurosurg. Psychiatry.

[31] Deters K.D., Risacher S.L., Farlow M.R., Unverzagt F.W., Kareken D.A., Hutchins G.D., Yoder K.K., Murrell J.R., Spina S., Epperson F. (2014). Cerebral hypometabolism and grey matter density in MAPT intron 10 +3 mutation carriers. Am. J. Neurodegener. Dis..

[32] Clarke M.T.M., St-Onge F., Beauregard J.M., Bocchetta M., Todd E., Cash D.M., Rohrer J.D., Laforce R. (2021). Early anterior cingulate involvement is seen in presymptomatic MAPT P301L mutation carriers. Alzheimer’s Res. Ther..

[33] Liu L., Chu M., Nie B., Liu L., Xie K., Cui Y., Kong Y., Chen Z., Nan H., Chen K. (2022). Reconfigured metabolism brain network in asymptomatic microtubule-associated protein tau mutation carriers: A graph theoretical analysis. Alzheimer’s Res. Ther..

[34] Chu M., Jiang D., Liu L., Nie B., Cui B., Wang Y., Rosa-Neto P., Wu L. (2023). Altered Anterior Insular Metabolic Connectivity in Asymptomatic MAPT P301L Carriers. J. Alzheimer’s Dis..

[35] Jacova C., Hsiung G.Y., Tawankanjanachot I., Dinelle K., McCormick S., Gonzalez M., Lee H., Sengdy P., Bouchard-Kerr P., Dinelle K. (2013). Anterior brain glucose hypometabolism predates dementia in progranulin mutation carriers. Neurology.

[36] Caroppo P., Habert M.O., Durrleman S., Funkiewiez A., Perlbarg V., Hahn V., Bertin H., Gaubert M., Routier A., Hannequin D. (2015). Lateral Temporal Lobe: An Early Imaging Marker of the Presymptomatic GRN Disease?. J. Alzheimer’s Dis..

[37] Saracino D., Sellami L., Boniface H., Houot M., Pélégrini-Issac M., Funkiewiez A., Rinaldi D., Locatelli M., Azuar C., Causse-Lemercier V. (2023). Brain Metabolic Profile in Presymptomatic GRN Carriers Throughout a 5-Year Follow-up. Neurology.

[38] Popuri K., Beg M.F., Lee H., Balachandar R., Wang L., Sossi V., Jacova C., Baker M., Shahinfard E., Pademakers R. (2021). FDG-PET in presymptomatic C9orf72 mutation carriers. NeuroImage Clin..

[39] De Vocht J., Blommaert J., Devrome M., Radwan A., Van Weehaeghe D., De Schaepdryver M., Ceccarini J., Rezaei A., Schramm G., van Aalst J. (2020). Use of Multimodal Imaging and Clinical Biomarkers in Presymptomatic Carriers of C9orf72 Repeat Expansion. JAMA Neurol..

[40] Greaves C.V., Rohrer J.D. (2019). An update on genetic frontotemporal dementia. J. Neurol..

[41] Titov D., Diehl-Schmid J., Shi K., Perneczky R., Zou N., Grimmer T., Li J., Drzezga A., Yakushev I. (2017). Metabolic connectivity for differential diagnosis of dementing disorders. J. Cereb. Blood Flow Metab..

[42] Filippi M., Agosta F., Scola E., Canu E., Magnani G., Marcone A., Valsasina P., Caso F., Copetti M., Comi G. (2013). Functional network connectivity in the behavioral variant of frontotemporal dementia. Cortex.

[43] Schimmelpfennig J., Topczewski J., Zajkowski W., Jankowiak-Siuda K. (2023). The role of the salience network in cognitive and affective deficits. Front. Hum. Neurosci..

[44] Ripp I., Stadhouders T., Savio A., Goldhardt O., Cabello J., Calhoun V., Riedl V., Hedderich D., Diehl-Schmid J., Grimmer T. (2020). Integrity of Neurocognitive Networks in Dementing Disorders as Measured with Simultaneous PET/Functional MRI. J. Nucl. Med..

[45] Malpetti M., Carli G., Sala A., Cerami C., Marcone A., Iannaccone S., Magnani G., Perani D. (2019). Variant-specific vulnerability in metabolic connectivity and resting-state networks in behavioural variant of frontotemporal dementia. Cortex.

[46] Rus T., Perovnik M., Vo A., Nguyen N., Tang C., Jamšek J., Popović K.Š., Grimmer T., Yakushev I., Diehl-Schmid J. (2023). Disease specific and nonspecific metabolic brain networks in behavioral variant of frontotemporal dementia. Hum. Brain Mapp..

[47] Nazem A., Tang C.C., Spetsieris P., Dresel C., Gordon M.L., Diehl-Schmid J., Grimmer T., Yakushev I., Mattis P.J., Ma Y. (2018). A multivariate metabolic imaging marker for behavioral variant frontotemporal dementia. Alzheimer’s Dement..

[48] Khokhar S.K., Kumar M., Arshad F., Goyal S., Tiwari M., Thanissery N., Ramakrishnan S., Nagaraj C., Kashyap R., Mangalore S. (2025). Multiplex connectomics reveal altered networks in frontotemporal dementia: A multisite study. Netw. Neurosci..

[49] Lanata S.C., Miller B.L. (2016). The behavioural variant frontotemporal dementia (bvFTD) syndrome in psychiatry. J. Neurol. Neurosurg. Psychiatry.

[50] Ducharme S., Price B.H., Larvie M., Dougherty D.D., Dickerson B.C. (2015). Clinical Approach to the Differential Diagnosis Between Behavioral Variant Frontotemporal Dementia and Primary Psychiatric Disorders. Am. J. Psychiatry.

[51] Tsoukra P., Velakoulis D., Wibawa P., Malpas C.B., Walterfang M., Evans A., Farrand S., Kelso W., Eratne D., Loi S.M. (2022). The Diagnostic Challenge of Young-Onset Dementia Syndromes and Primary Psychiatric Diseases: Results From a Retrospective 20-Year Cross-Sectional Study. J. Neuropsychiatry Clin. Neurosci..

[52] Zapata-Restrepo L., Rivas J., Miranda C., Miller B.L., Ibanez A., Allen I.E., Possin K. (2021). The Psychiatric Misdiagnosis of Behavioral Variant Frontotemporal Dementia in a Colombian Sample. Front. Neurol..

[53] van Vliet D., de Vugt M.E., Bakker C., Pijnenburg Y.A., Vernooij-Dassen M.J., Koopmans R.T., Verhey F.R.J. (2013). Time to diagnosis in young-onset dementia as compared with late-onset dementia. Psychol. Med..

[54] de Boer S.C.M., Gossink F., Krudop W., Vijverberg E., Schouws S., Reus L.M., Pijnenburg Y.A.L., Dols A. (2023). Diagnostic Instability Over Time in the Late-Onset Frontal Lobe Syndrome: When Can We Say it’s FTD?. Am. J. Geriatr. Psychiatry.

[55] Ducharme S., Dols A., Laforce R., Devenney E., Kumfor F., van den Stock J., Dallaire-Théroux C., Seelaar H., Gossink F., Vijverberg E. (2020). Recommendations to distinguish behavioural variant frontotemporal dementia from psychiatric disorders. Brain.

[56] van Engelen M.E., Verfaillie S.C.J., Dols A., Oudega M.L., Boellaard R., Golla S.S.V., den Hollander M., Ossenkoppele R., Scheltens P., van Berckel B.N.M. (2023). Altered brain metabolism in frontotemporal dementia and psychiatric disorders: Involvement of the anterior cingulate cortex. EJNMMI Res..

[57] Devenney E., Swinn T., Mioshi E., Hornberger M., Dawson K.E., Mead S., Rowe J.B., Hodges J.R. (2018). The behavioural variant frontotemporal dementia phenocopy syndrome is a distinct entity—evidence from a longitudinal study. BMC Neurol..

[58] Eratne D., Loi S.M., Walia N., Farrand S., Li Q.X., Varghese S., Walterfang M., Evans A., Mocellin R., Dhiman K. (2020). A pilot study of the utility of cerebrospinal fluid neurofilament light chain in differentiating neurodegenerative from psychiatric disorders: A ‘C-reactive protein’ for psychiatrists and neurologists?. Aust. N. Z. J. Psychiatry.

[59] Vijverberg E.G., Dols A., Krudop W.A., Del Campo Milan M., Kerssens C.J., Gossink F., Prins N.D., Stek M.L., Scheltens P., Teunissen C.E. (2017). Cerebrospinal fluid biomarker examination as a tool to discriminate behavioral variant frontotemporal dementia from primary psychiatric disorders. Alzheimer’s Dement..

[60] Katisko K., Cajanus A., Jääskeläinen O., Kontkanen A., Hartikainen P., Korhonen V.E., Helisalmi S., Haapasalo A., Koivumaa-Honkanen H., Herukka S.-K. (2020). Serum neurofilament light chain is a discriminative biomarker between frontotemporal lobar degeneration and primary psychiatric disorders. J. Neurol..

[61] Ducharme S., Bajestan S., Dickerson B.C., Voon V. (2017). Psychiatric Presentations of C9orf72 Mutation: What Are the Diagnostic Implications for Clinicians?. J. Neuropsychiatry Clin. Neurosci..

[62] Vijverberg E.G., Wattjes M.P., Dols A., Krudop W.A., Möller C., Peters A., Kerssens C.J., Gossink F., Prins N.D., Stek M.L. (2016). Diagnostic Accuracy of MRI and Additional [18F]FDG-PET for Behavioral Variant Frontotemporal Dementia in Patients with Late Onset Behavioral Changes. J. Alzheimer’s Dis..

[63] Hellwig S., Frings L., Heibel M., Schroeter N., Blazhenets G., Domschke K., Brumberg J., Meyer P.T. (2025). [(18)F]Fluorodeoxyglucose position emission tomography for differential diagnosis of depressive cognitive impairment: Incremental value compared with clinical diagnosis. Br. J. Psychiatry.

[64] Neary D., Snowden J.S., Gustafson L., Passant U., Stuss D., Black S., Freedman M., Kertesz A., Robert P.H., Albert M. (1998). Frontotemporal lobar degeneration: A consensus on clinical diagnostic criteria. Neurology.

[65] Delvecchio G., Mandolini G.M., Arighi A., Prunas C., Mauri C.M., Pietroboni A.M., Marotta G., Cinnante C.M., Triulzi F.M., Galimberti D. (2019). Structural and metabolic cerebral alterations between elderly bipolar disorder and behavioural variant frontotemporal dementia: A combined MRI-PET study. Aust. N. Z. J. Psychiatry.

[66] Ishii K., Sakamoto S., Sasaki M., Kitagaki H., Yamaji S., Hashimoto M., Imamura T., Shimomura T., Hirono N., Mori E. (1998). Cerebral glucose metabolism in patients with frontotemporal dementia. J. Nucl. Med..

[67] Xia Y., Lu S., Wen L., Eberl S., Fulham M., Feng D.D. (2014). Automated identification of dementia using FDG-PET imaging. BioMed Res. Int..

[68] Gjerum L., Frederiksen K.S., Henriksen O.M., Law I., Bruun M., Simonsen A.H., Mecocci P., Baroni M., Dottorini M.E., Koikkalainen J. (2020). Evaluating 2-[(18)F]FDG-PET in differential diagnosis of dementia using a data-driven decision model. NeuroImage Clin..

[69] Perovnik M., Vo A., Nguyen N., Jamšek J., Rus T., Tang C.C., Trošt M., Eidelberg D. (2022). Automated differential diagnosis of dementia syndromes using FDG PET and machine learning. Front. Aging Neurosci..

[70] Barnard L., Botha H., Corriveau-Lecavalier N., Graff-Radford J., Dicks E., Gogineni V., Zhang G., Burkett B.J., Johnson D.R., Huls S.J. (2025). An FDG-PET-Based Machine Learning Framework to Support Neurologic Decision-Making in Alzheimer Disease and Related Disorders. Neurology.

